# *Asaia* Activates Immune Genes in Mosquito Eliciting an Anti-*Plasmodium* Response: Implications in Malaria Control

**DOI:** 10.3389/fgene.2019.00836

**Published:** 2019-09-25

**Authors:** Alessia Cappelli, Claudia Damiani, Maria Vittoria Mancini, Matteo Valzano, Paolo Rossi, Aurelio Serrao, Irene Ricci, Guido Favia

**Affiliations:** School of Biosciences & Veterinary Medicine, University of Camerino, Camerino, Italy

**Keywords:** *Asaia*, *Plasmodium*, malaria, symbiotic control, immune system

## Abstract

In mosquitoes, the discovery of the numerous interactions between components of the microbiota and the host immune response opens up the attractive possibility of the development of novel control strategies against mosquito borne diseases. We have focused our attention to *Asaia*, a symbiont of several mosquito vectors who has been proposed as one of the most potential tool for paratransgenic applications; although being extensively characterized, its interactions with the mosquito immune system has never been investigated. Here we report a study aimed at describing the interactions between *Asaia* and the immune system of two vectors of malaria, *Anophelesstephensi* and *An. gambiae*. The introduction of *Asaia* isolates induced the activation of the basal level of mosquito immunity and lower the development of malaria parasite in *An. stephensi*. These findings confirm and expand the potential of *Asaia* in mosquito borne diseases control, not only through paratransgenesis, but also as a natural effector for mosquito immune priming.

## Introduction

Malaria is a mosquito-borne disease (MBD) caused by a parasite of the genus *Plasmodium*, responsible of about five hundred thousand deaths per year (World Health Organization, 2018). Considering the lack of an effective vaccine, chemical insecticides, applied either as Indoor Residual Spraying (IRSs) or Insecticide Treated Nets (ITNs), are the main prevention tools currently adopted (World Health Organization, 2014). Nevertheless, these preventive methods are losing their effectiveness due to onset of resistance phenomena in vector populations. Moreover, their massive use is highly toxic to both humans and the environment. During the last decades, new strategies have been developed to tackle malaria and more generally MBD’s. In particular, some studies have converged into the development of strategies known as Symbiotic Control (SC), using the symbiotic microorganisms colonizing vector hosts to combat the development of the parasite within them or to interfere with their competence and fitness (Ricci et al., 2012).

In mosquitoes, the malaria parasite completes its life-cycle starting from the ingestion of thousand gametocytes during the infected blood meal. Although thousands of gametocytes are ingested, only a small portion (about 10%) develops into ookinetes, and of these about 5% crosses the midgut epithelium to form the oocysts (Wang and Jacobs-Lorena, 2013). Nevertheless, an amplification of the parasites number occurs when the oocysts form and release thousands of sporozoites in the hemocoel that invade the salivary glands and will be injected in a next individual through the mosquito bite. The replication bottleneck occurring in the midgut is mainly due to two different mechanisms of mosquito innate immune system: (i) a humoral response involving a complement-like system and the transcriptional up-regulation of antimicrobial peptides (AMPs) and other immune effectors and (ii) a cell-mediated response, included phagocytosis and/or melanization (Clayton et al., 2014). The drastic reduction in numbers of the parasite makes this compartment, an ideal target to interfere with *Plasmodium* development in the mosquito.

Moreover, the direct interactions between gut microbiota and malaria parasite have been largely documented (Dong et al., 2009). In fact, bacteria can inhibit *Plasmodium* development producing a physical barrier that hinders the interaction between ookinetes and the midgut epithelium, or through the production of enzymes and toxins (Azambuja et al., 2005). Alternatively, components of the mosquito microbiota can indirectly cause alterations of the insect physiology inducing the activation of innate immune responses that are cross-reactive between bacteria and parasites, and adversely affect pathogen infection (Dong et al., 2009). In addition, the natural microbiota was proved to be involved in the inhibition of other pathogenic organisms in different mosquito species: bacteria of *Aedes aegypti* are able to induce basal-level immunity inhibiting dengue virus infection (Xi et al., 2008) and antibiotic-treated *Culex bitaeniorhynchus* showed higher susceptibility to the Japanese encephalitis virus (Mourya and Soman, 1985).

In light of these considerations, the objective of this study was the investigation of the immune response in *Anopheles stephensi* and *An. gambiae* mosquitoes challenged with *Asaia* sp.

*Asaia* is a symbiont of several mosquito species and it has characterized as a potential candidate for SC interventions, like paratransgensis. The features of this bacteria supporting its use in vector control applications are: i) a strict and conserved association with the mosquito host; ii) vertical and horizontal transmission routes; iii) easy cultivability and genetic transformability with exogenous DNA (Favia et al., 2007; Damiani et al., 2008; Damiani et al., 2010). Moreover, a strain of *Asaia* isolated from *An. stephensi*, has been transformed to express and secrete anti-*Plasmodium* molecules able to interference activity against *Plasmodium berghei* resulting in a significant reduction of oocysts development (Bongio and Lampe, 2015; Shane et al., 2018). Although *Asaia* has been characterized as a potential tool for its applications, its interactions with the mosquito immune system has never been investigated. This study aims at defining the role of this symbiont on the modulation of gene expression of mosquito immunity effectors and its effect on the progression of *P. berghei* infection within two of the main malaria vectors in Asia and Africa.

## Materials and Methods

### Mosquitoes

*An. stephensi* (Liston strain) and *An. gambiae* (G3 strain) were reared at standard laboratory conditions, at 29°C and 85% ± 5 relative humidity with photoperiods (12:12 Light–dark). Unless otherwise specified, adult insects were maintained with 5% sucrose solution *ad libitum*, and adult females were fed on mouse blood for egg laying. Larvae were maintained in spring water and fed daily with commercial fish food.

### *Asaia* Cultures

The *Asaia* SF2.1 strain isolated from *An. stephensi* (Favia et al., 2007) was used for mosquito colonization. Bacteria were grown 24 h at 30°C in GLY medium (25 g/L glycerol, 10 g/L yeast extract, pH 5). Two different concentrations were prepared from an overnight culture: (i) 10^8^ cells/ml and (ii) 10^4^ cells/ml. Dilutions were washed three times with 1 vol of 0.9% NaCl solution, centrifuged at 4,300 rpm for 10 min and then, re-suspended in 1 vol of 5% (wt/vol) sterile sucrose solution. The concentration of the cell suspensions was validated prior mosquito colonization by plating scalar dilutions on Gly agar medium (25 g/L glycerol, 10 g/L yeast extract, 20 g/L agar, pH 5) and by calculating CFU/ml.

### *Plasmodium berghei* Infection

*Pb*GFP_CON_, a GFP-tagged recombinant strain that constitutively expresses GFP at higher levels throughout the complete life cycle from an integrated transgene, was used for mosquito challenges (Franke-Fayard et al., 2004). Infected blood meals were performed using mice showing a parasitemia around 3–5% (Sinden, 1997) in a chamber at 20°C and 95± 5% humidity, a step strongly required for parasite development in laboratory conditions (Capone et al., 2013). Briefly, 8-week-old female mice were infected with *P. berghei* PbGFP_CON_ by acyclic passages through an intraperitoneal injection of blood from the tail vein of an infected mouse with around 3–5% parasitemia. Infected mice were monitored every couple of days for parasitemia by fluorescent microscopy as well as gametocytemia evaluation by through Giemsa-stained blood smear.

### Experimental Design

**Figure 1:**
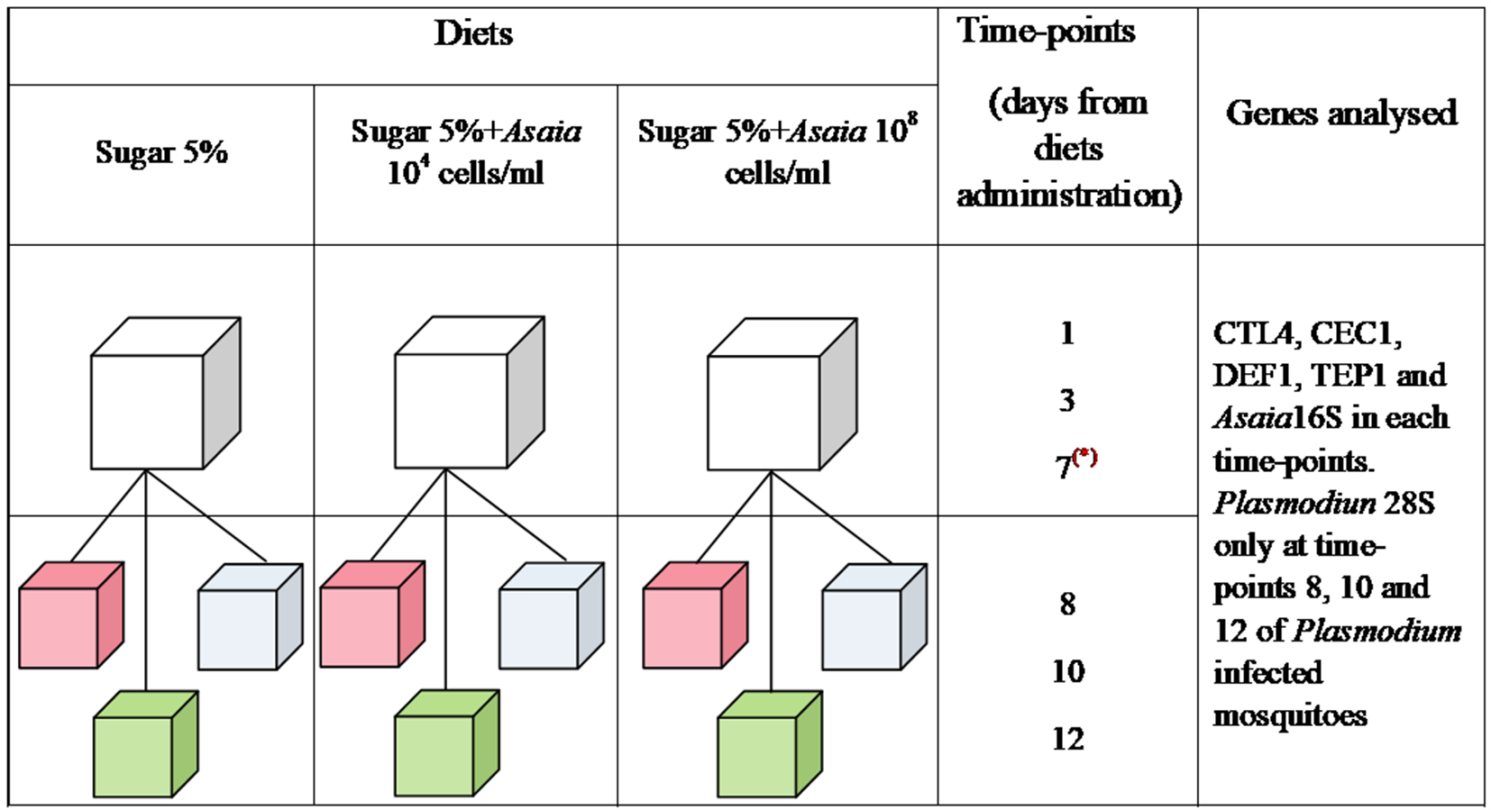
Schematic representation of the experimental design. Three groups of mosquitoes were fed from the emergence for 7 days with different diets: Sugar 5%, Sugar 5% + *Asaia* 10^4^ cells/ml and Sugar 5% + *Asaia* 10^8^ cells/ml. At day 7 (represented by a red asterisk), 70 mosquitoes were split in three further cages and, at the same day, three different treatments were administrated: uninfected blood meal (pink), infected blood meal (green) and sugar diet (cyan). In each time-points, *Asaia* density and the expression level of *CEC1*, *DEF1*, *CTL4* and *TEP1* genes were evaluated. Moreover, *Plasmodium* load was assessed in mosquitoes infected with *P. berghei* at time-point 8, 10 and 12. The asterisk indicates the day of blood meal.

Three groups were set up with 250 female pupae each. After emerging, mosquitoes were fed with three different diets: sterile sugar solution, sugar solution supplemented with 10^4^ cells/ml of *Asaia*, and sugar solution with 10^8^ cells/ml of *Asaia*. The three mosquito cohorts fed on different diets were named Sugar, Asa4, and Asa8, respectively (**Figure 1**). The analysis of immune genes expression and *Asaia* density were conducted on 12 mosquitoes for each treatment at different time-points: 1, 3, and 7 days post-emergence. At day 7, 70 mosquitoes from each group were split into three further groups: uninfected blood meal, *Plasmodium*-infected blood meal, and a control with constant sugar supply. Only fully engorged females were considered for further analyses. At day 8, 10 and 12 post-emergence, 9 mosquitoes from each group were sampled for further analyses.

Two experimental replicates were performed and the results shown represent the average of both experiments. The experimental design and groups set-up is summarised in **Figure 1**.

The establishment of an *Asaia*-aposymbiotic population was prevented by the significantly reduced life span of antibiotic treated mosquitoes.

### RNA Extraction and cDNA Synthesis.

Total RNA was extracted from single mosquitoes using RNAzol reagent (Sigma-Aldrich USA), according to the manufacturer’s instructions. cDNA was generated by reverse transcription of 1µg of total RNA using PrimeScript RT Reagent Kit (Takara, USA).

### Quantitative RT-PCR

Expression of anti-microbial peptide cecropin (*CEC1*) and defensin (*DEF1*), C-type lectin 4 (*CTL4*) and Thioester-containing protein 1 (*TEP1*) was assessed in all mosquito cohorts. Moreover, *Asaia* density was evaluated for every group, whilst *Plasmodium* loads for infected mosquitoes at three different time points. The PCRs reaction included 1X SybrGreen Master Mix (Fermentas, Lithuania), 200 nM of oligonucleotides and 2µl of cDNA. Oligonucleotide sequences and their efficiency are summarized in **Table S1**. The efficiency of each primer set was calculated by the amplification of eight serial dilutions of cDNA.

Reactions were run on a CFX thermocycler (Bio-Rad, USA) using the following cycling conditions: 1 cycle of 95°C for 10 min, 40 -cycles of 95°C for 1 min, 60°C for 1 min, and 74°C for 30 s, with the exception of the *Plasmodium*-specific set whose annealing temperature was 58°C as described before (Jaramillo-Gutierrez et al., 2009).

Gene expression levels of target genes were normalized against the internal species-specific reference ribosomal gene RpS7 for *An. stephensi* and *An. gambiae*. The relative expression of immune gene respect to control group (Sugar) was calculated using the Livak Method (Livak and Schmittgen, 2001).

### Statistical Analysis

Statistical analysis was performed using the Bio-Rad CFX Manager Software and the GraphPad software (http://www.graphpad.com). For each group, mean values from the biological replicates for each time-point were taken into account and the standard error (SEM) was calculated. One-way ANOVA test and the post-hoc test Dunn were used to assess the statistical differences between gene expressions in *Asaia*-challenged *An. stephensi* and *An. gambiae* mosquitoes and the presence of *Plasmodium* between treatments and controls. Differences in *Asaia* density among treatments over time and the gene expression in *Asaia*-challenged mosquitoes after uninfected or infected blood meal were calculated by two- way ANOVA test and the Bonferroni post-hoc test.

### Microbiota Analysis

16S Miseq analysis was conducted on cDNA of *An. stephensi* mosquitoes fed on different diets by LGC Genomics (Berlin, Germany). The RNA came from the same mosquito described for the quantitative RT-PCR experiment. Briefly, the cDNA was amplified using the primers 341F and 785R targeting the region V3–V4 of 16S ribosomal RNA (Klindworth et al., 2013). Quality control of raw data was done using Mothur (Schloss et al., 2009) and then the sequences were searched for matching in the SILVA taxonomy database. OTUs diversity analysis was performed using QIIME software (Caporaso et al., 2010).

## Results

### *Asaia* Colonization Activates the Basal Immune Levels in *An. stephensi* and *An. gambiae*

**Figure 2:**
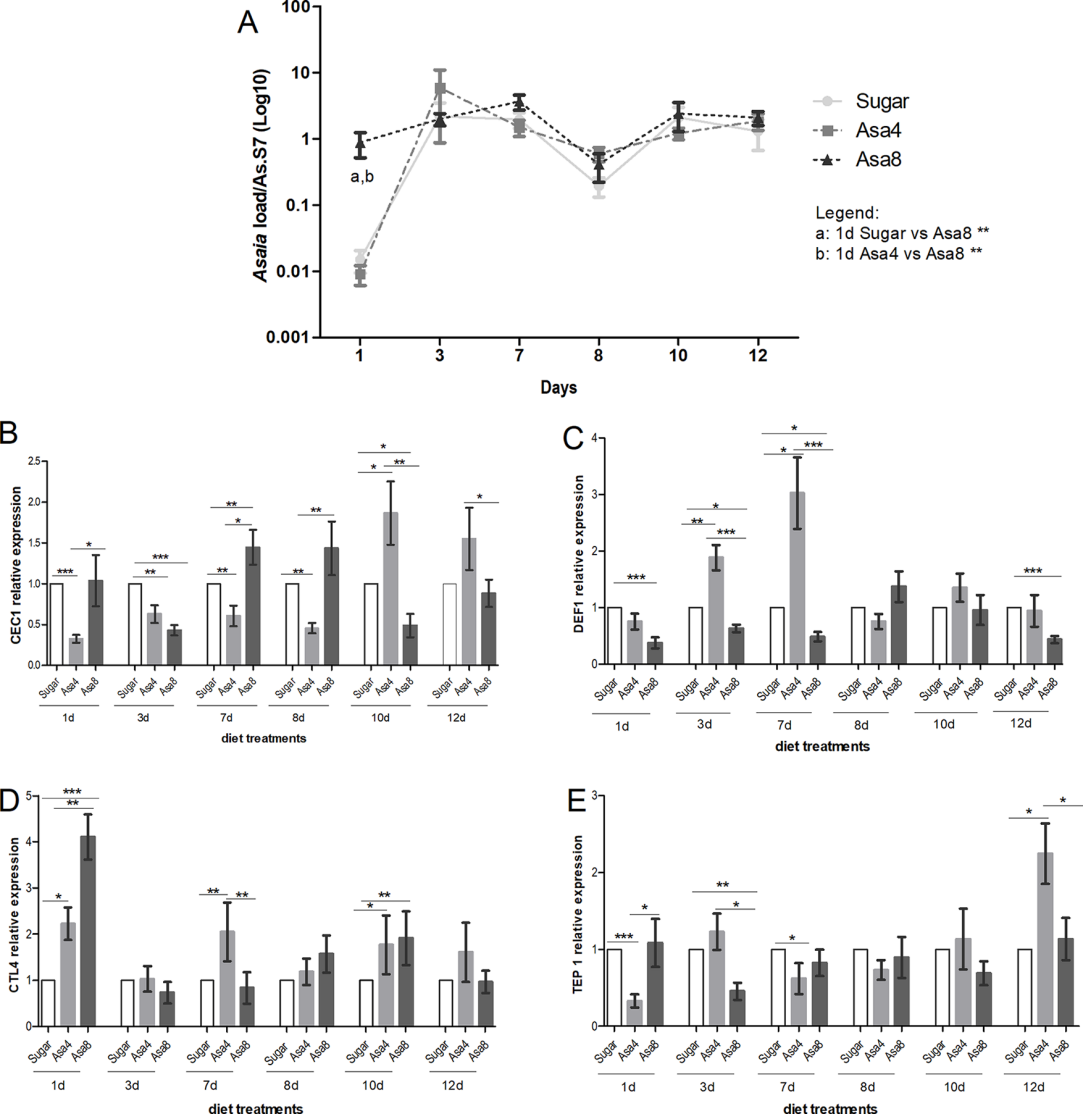
Gene expression and *Asaia* load in *An. stephensi*. Evaluation of *Asaia* load **(A)** at different time points and *CEC1* **(B)**, *DEF1* **(C)**, *CTL4* **(D)**, and *TEP1* **(E)** genes expression. *Asaia* density **(A)** was normalized on RpS7 as a reference gene. The relative expression of *CEC1*, *DEF1*, *CTL4*, and *TEP1* was normalized on RpS7 and compared to a calibrator (sugar group). Values represent the average ± SEM from two biological replicates. Differences between *Asaia* load in the groups were calculated by two-way ANOVA test and the Bonferroni post-hoc test. One-way ANOVA test and Dunn post-hoc test were used to calculate statistics between genes expression.*p < 0.05, **p < 0.01, ***p < 0.001.

Despite the different concentrations of bacteria provided in the diet, *Asaia* quickly reached a similar homeostasis within 3 days in *An. stephensi*, where this bacterial species has been described as one of the main component of its natural microbiota (**Figure 2**). At the first time-point (1 day) sugar-fed and Asa4-fed mosquitoes showed similar *Asaia* density, compared to that detected in the cohort fed with Asa8 (p < 0.01): this early time-point is likely to reflect the quantity of *Asaia* administrated with the diets (**Figure 2A**). Between days 1 and 3, an increasing replication of *Asaia* in the control and Asa4-mosquitoes was observed, while Asa8-individuals showed constant bacterial density. At day 3, *Asaia* reached a similar homeostasis in all treatments, which was maintained during the following time-points, suggesting both adaptation of the bacterium and tolerance of the host (**Figure 2A**).

For each time-point, the expression of *CEC1*, *DEF1*, *CTL4*, and *TEP1* genes was assessed (**Figures 2 B–E**). After 24 h from *Asaia* administration, the expression of *CTL4* resulted significantly up-regulated in Asa4 (p < 0.05) and Asa8 mosquitoes (p < 0.001) with respect to the control, and between the two concentrations of administrated *Asaia* (p < 0.01) (**Figure 2D**). Expression of the two analyzed AMPs appears to be differentially regulated. *DEF1* expression is elicited by the lower concentration of *Asaia* reaching its maximal up-regulation at day 7, which coincides with the initial exponential phase of bacterial replication (**Figure 2C**). On the contrary, supplementation of higher concentrations of bacteria down-regulates *DEF1* expression during the initial time points (**Figure 2C**).

*CEC1* showed a delayed activation at day 10 after an initial down-regulation in Asa4-challenged samples. In contrast, higher doses of *Asaia* appeared to induce an earlier up-regulation of *CEC1* between days 7 and 8, followed by its down-regulation from day 10 (**Figure 2B**). The *TEP1* seemed to be marginally involved in the immune response against supplemented *Asaia* during the time window analyzed, except for being up-regulated in the Asa4 group after 12 days (**Figure 2E**).

**Figure 3:**
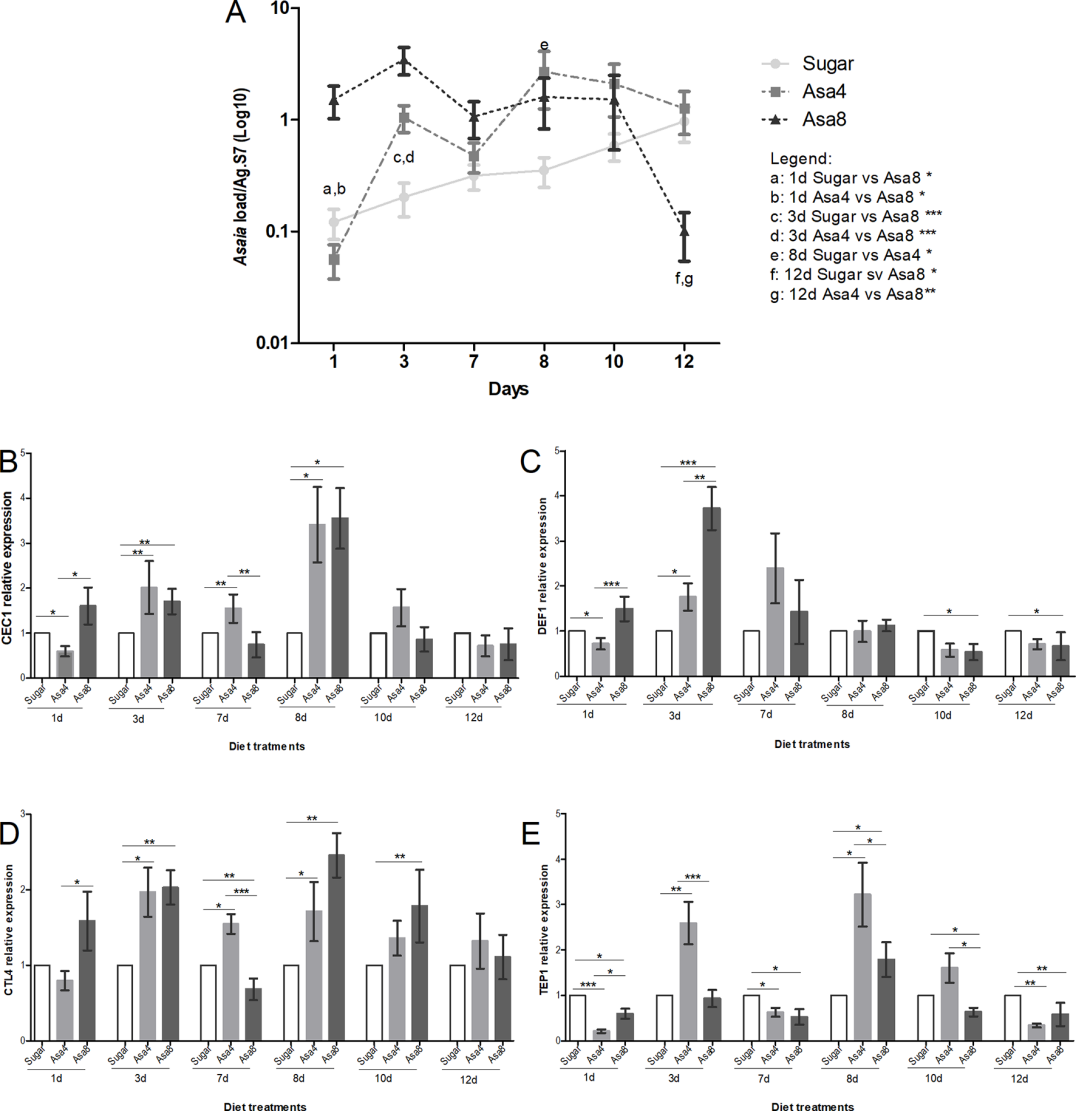
Gene expression and *Asaia* load in *An. gambiae*. Evaluation of *Asaia* load **(A)** at different time points and *CEC1* **(B)**, *DEF1* **(C)**, *CTL4* **(D)**, and *TEP1* **(E)** genes expression. *Asaia* density **(A)** was normalized on RpS7 as a reference gene. The relative expression of *CEC1*, *DEF1*, *CTL4*, and *TEP1* was normalized on RpS7 and compared to a calibrator (sugar group). Values represent the average ± SEM from two biological replicates. Differences between *Asaia* load in the groups were calculated by two-way ANOVA test and the Bonferroni post-hoc test. One-way ANOVA test and Dunn post-hoc test were used to calculate statistics between genes expression.*p < 0.05, **p < 0.01, ***p < 0.001.

Similarly to *An. stephensi*, females of *An. gambiae* were orally fed with the same concentrations of *Asaia* cells. Consistently with its role as a secondary component of the natural *An. gambiae* microbiota, *Asaia* doses are differently tolerated and regulated within the African malaria vector. A comparable homeostasis between doses is reached only after 7 days. Between 3 and 7 days, differences between bacteria-challenged mosquitoes appeared to be controlled and similarly stabilized: the persistence of *Asaia* in challenged individuals at the higher concentrations, compared to sugar-fed mosquitoes is maintained until the last time point where Asa4-fed mosquitoes mirrored the natural trend of sugar-fed individuals, whilst Asa8-colonized individuals experienced a significant drop in *Asaia* density (**Figure 3A**).

Interestingly, in individuals of the group Asa8, the synergistic up-regulation of *CEC1*, *DEF1*, and *CTL4* genes was observed during the initial time-points, probably elicited by the substantial colonization of the introduced bacteria (**Figures 3B–D**). In particular, *CEC1* resulted to be initially up-regulated with respect to both groups (**Figure 3B**). *DEF1* gene expression is characterized by a significant initial up-regulation during the early infection phase compared to the other groups (**Figure 3C**). Similarly, an up-regulation was detected in *CTL4* expression level at the 1 day and 3, which, unlikely the AMPs expression pattern, resumed and persisted until day 10 (**Figure 3D**). The involvement of the effector of the complement system, *TEP1*, seemed to be limited to a minimal up-regulation on day 8 when compared to sugar-fed controls (p < 0.05) (**Figure 3E**).

In Asa4-challenged mosquitoes, the mounting of the immune response appeared delayed, starting after 3 days from *Asaia* administration, coinciding with the exponential bacterial replication at this time point (**Figures 3B–E**). The transcriptional activation of the two AMPs appeared to be similarly regulated during the initial infection phase, after which *CEC1* up-regulation is observed (**Figure 3B**). Alike Asa8, *CTL4* maintained its up-regulation from day 3 to day 8 compared to the sugar control (p < 0.05) in each time-point (**Figure 3D**). *TEP1* resulted repetitively up-regulated compared to the control at day 3 (p < 0.01) and day 8 (p < 0.05), coinciding with the fluctuations in *Asaia* density.

### *Asaia*-Induced Immune Response Reduces *P. berghei in An. stephensi*, but Not in *An. gambiae*

**Figure 4:**
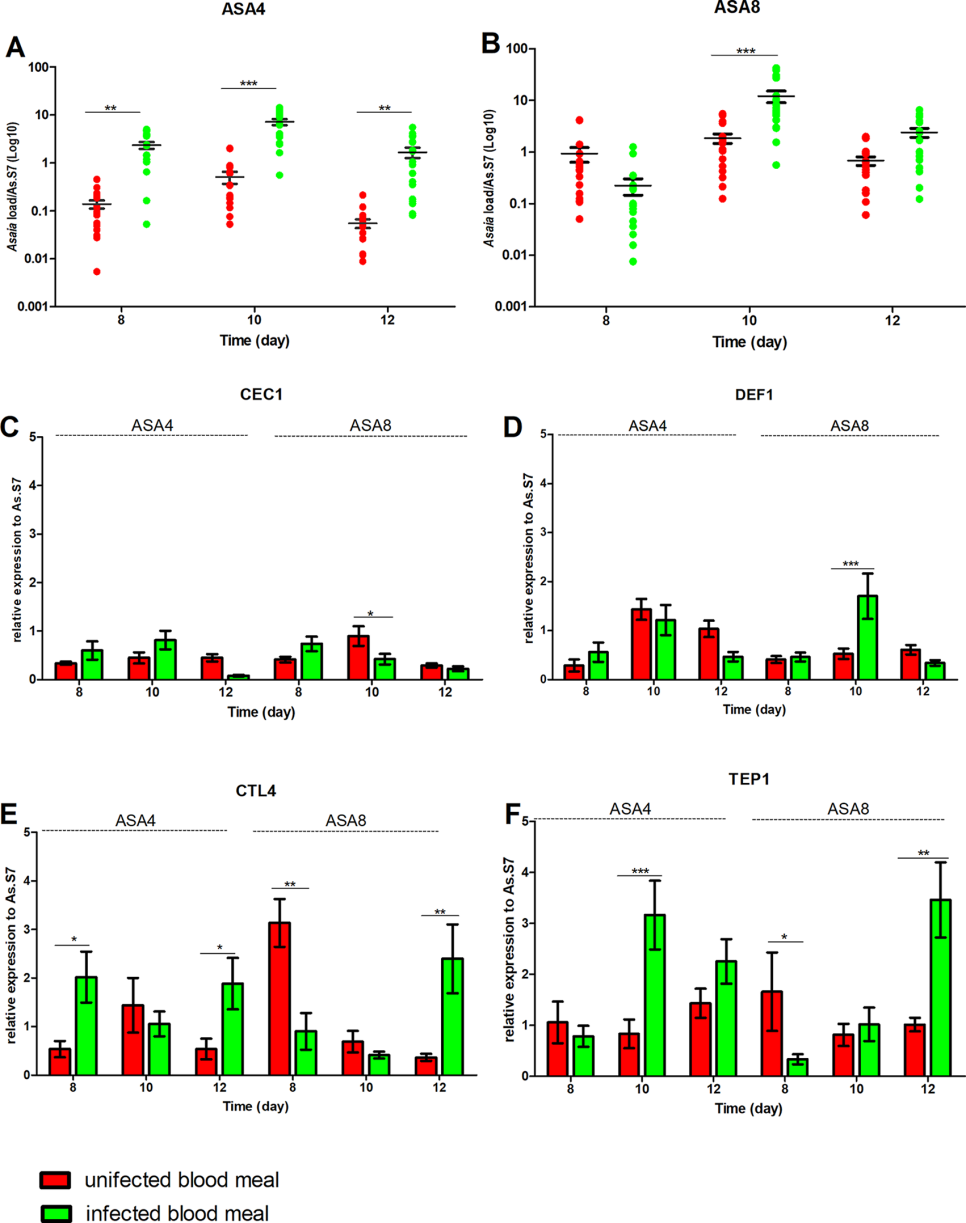
Effect of uninfected and *P. berghei*-infected blood meal on *Asaia* density and immune genes expression in *An. stephensi*. Evaluation of the fluctuations of *Asaia* density in mosquitoes challenged with the two doses of Asa4 **(A)** and Asa8 **(B)** and the related modulation of immune effectors after uninfected and infected blood meal **(C**, **D**, **E**, and **F)**. Shown values represent the relative genes expression normalized on the reference gene RpS7 and against the calibrator represented by sugar-fed controls (relative expression of sugar-fed mosquitoes in **Figure S1-A**). The statistical differences of *Asaia* density and the expression level of the genes in the groups were calculated by two-way ANOVA test and the Bonferroni post-hoc test. *p < 0.05, **p < 0.01, ***p < 0.001.

**Figure 5:**
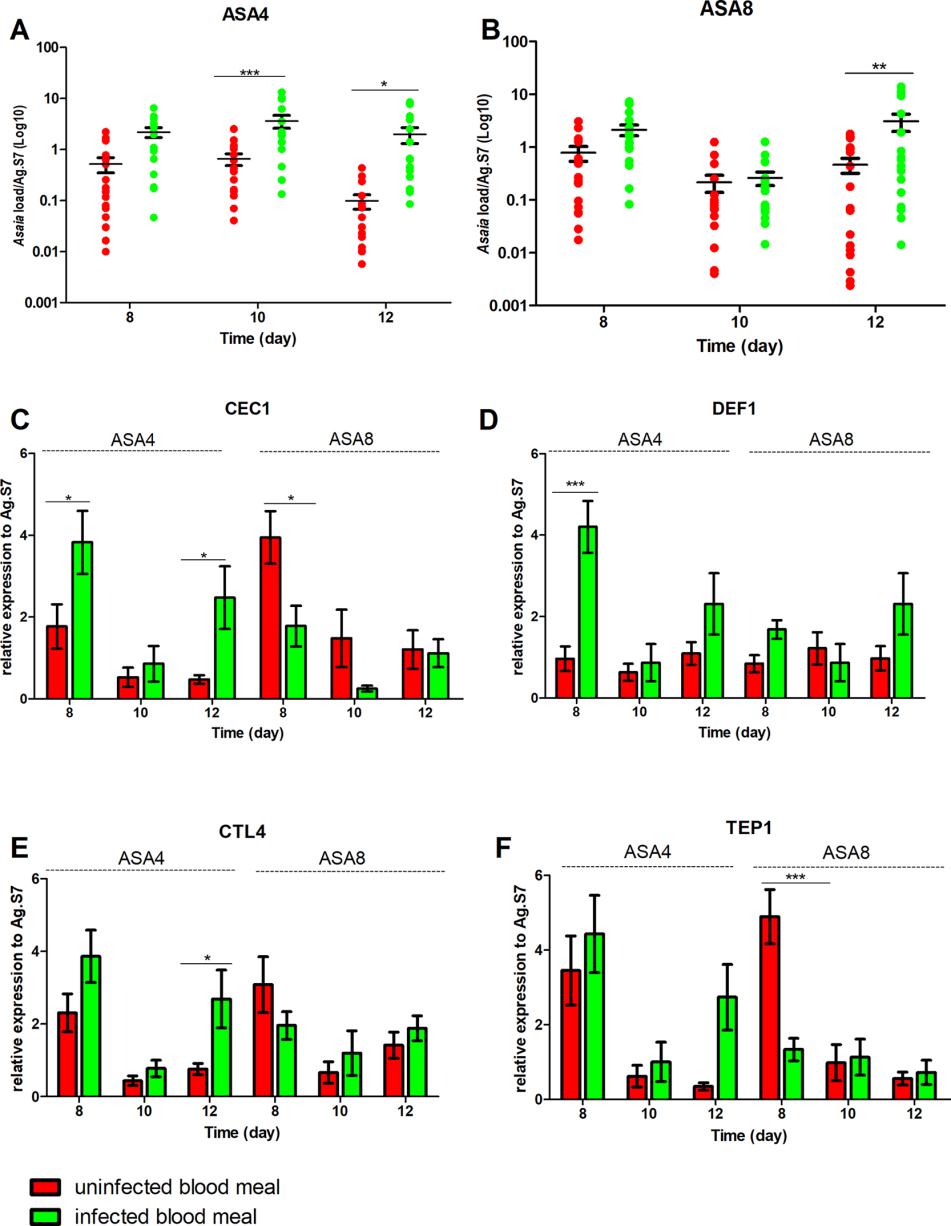
Effect of uninfected and *P. berghei*-infected blood meal on *Asaia* density and immune genes expression in *An. gambiae*. Evaluation of the fluctuations of *Asaia* density in mosquitoes challenged with two doses of *Asaia* cells: Asa4 **(A)** and Asa8 **(B)**, and the related modulation of immune effector genes after an uninfected and an infected blood meal **(C**, **D**, **E**, **F)**. Shown values represent the relative genes expression normalized on the reference gene RpS7 and against the calibrator represented by sugar-fed controls (relative expression of sugar-fed mosquitoes in **Figure S1-B**). The statistical differences between *Asaia* density and the expression level of genes in the groups were calculated by two-way ANOVA test with Bonferroni post-hoc test. *p < 0.05, **p < 0.01, ***p < 0.001.

After 7 days of bacterial exposure, mosquitoes were blood fed with uninfected and *P. berghei*- infected blood, and the transcriptional activation of target immune genes was evaluated (**Figures 4** and **5**). In both mosquito species, the presence of *Plasmodium* in the blood meal of Asa4-challenged mosquitoes induced an increase in *Asaia* replication: in *An. stephensi* a peak in density was detected after 24 h (**Figure 4A**), while in An. *gambiae* is looks delayed (**Figure 5A**). When higher concentrations of *Asaia* are introduced (Asa8), a similar delay between mosquito species is observed (**Figures 4B** and **5B**).

In *An. stephensi*, populations treated with *Asaia*-enriched diets do not show any particular activation of AMPs (**Figures 4C, D**) while the time window between days 10 and 12 coincides with the significant up-regulation of *TEP1*, often combined with *CTL4*, with both concentrations of *Asaia*, after an infected blood meal (**Figures 4E, F**).

The gene expression profiles for *An. gambiae* challenge with Asa4 revealed a very early activation at day 8, meaning 24 h after the blood meal, of *CEC1* and *DEF1*, in *Plasmodium*-infected mosquitoes (**Figures 5C, D**). At the time point, in *Plasmodium*-infected Asa8-mosquitoes, do not show any particular activation of AMPs (**Figures 5C, D**). Interestingly, no up-regulation of *TEP1* expression of infected-mosquitoes has been observed in both concentrations. Only *CTL4* showed higher expression in infected mosquitoes challenged with Asa4 after 12 days (**Figures 5E, F**).

**Figure 6:**
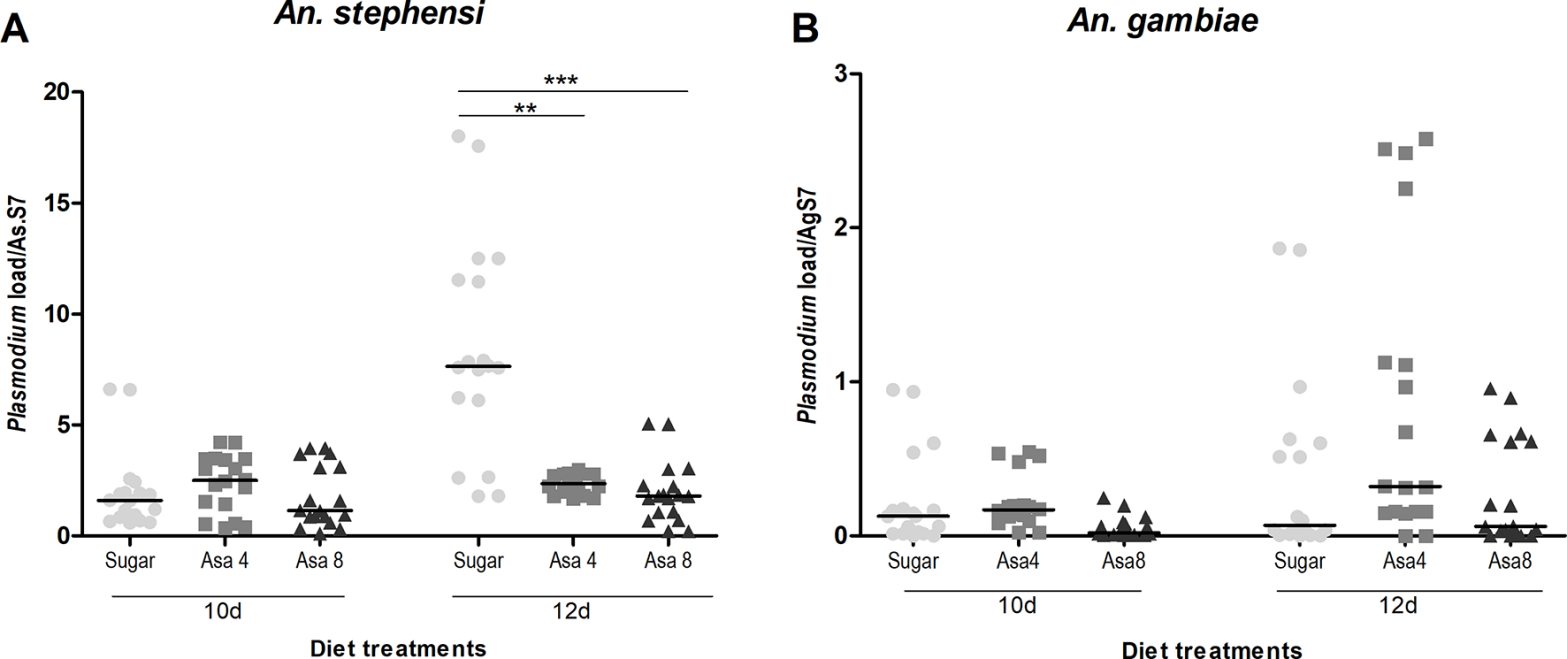
*Plasmodium* load in *An. stephensi* and *An. gambiae* mosquitoes challenged with *Asaia*. Evaluation of *Plasmodium* load has been performed at two time-points (three and five days after infected blood meal) in both *An. stephensi* **(A)** and *An. gambiae* **(B)**. In the y axes the relative abundance of *Plasmodium* compared to the reference gene RpS7 is reported. Symbols in the plot represent individual mosquitoes (n = 9 per group of two experimental replicates, in total 18 mosquitoes) with horizontal lines indicating the medians (m). Statistical analysis was calculated with One-way ANOVA test and Dunn post-hoc test. **p < 0.01; ***p < 0.001.

The hypothesis of a correlation between *Plasmodium* development and the colonization of *Asaia* at different concentrations was evaluated. Interestingly, in *An. stephensi* a significant reduction of *Plasmodium* ribosomal gene expression was detected in both treated groups (Asa4 and Asa8 groups) after 12 days (**Figure 6A**). The presence of the malaria parasite was also evaluated in *An. gambiae*: quantification of the parasite showed no significant reduction in *Plasmodium* replication, despite the activation of several immune genes (**Figure 6B**).

### *An. stephensi* Microbiota Abundance

The microbiota composition of *An. stephensi* fed on different diets was analyzed in order to directly correlate the immune response to a massive *Asaia* presence, especially in *P. berghei* infected mosquitoes where a high reduction of oocyst development was observed in mosquitoes infected with *Asaia*. At phylum level, Proteobacteria was the main predominant phylum present in all samples. Actinobacteria and Firmicutes were abundant in Sugar and Asa4 groups at 1 day and in 3 day only (**Figure S2**). *Asaia* represented the dominant genus of Proteobacteria phylum (**Figure S3**), reaching early the highest percentage in abundance in the mosquito populations that have been administrated with *Asaia*-enriched diets. After infected or uninfected blood meal, *Asaia* was the dominant bacteria in the mosquito populations with an OTUs abundance range between 70–90%.

## Discussion

The mosquito gut microbiota plays a crucial role in the host physiology contributing to the maintenance of metabolism and immunity homeostasis, but it can also stimulate a basal immune activity impacting on mosquito’s vector capacity (Dong et al., 2009). In fact, the introduction of special bacterial isolates in mosquitoes are able to trigger their innate immune response, which correlates with a decrease in the transmission of the malaria parasite (Frolet et al., 2006).

Although *Asaia* has already been described as able to stimulate the expression of some AMPs in mosquito while not being affected by phagocytosis (Capone et al., 2013), our results detailed the interactions between this bacteria and the mosquito immune system. We focused on some genes involved in *Plasmodium* surveillance: i) *CEC1* and *DEF1*, belonging to AMPs gene families, are involved in the elimination of viruses, bacteria and *Plasmodium* (Bartholomay et al., 2004; Xi et al., 2008); ii) *CTL4*, encodes for C-type lectins that control the microbiota homeostasis (Hillyer, 2010; Pang et al., 2016); iii) *TEP1*, mainly involved in *Plasmodium* killing (Blandin et al., 2004; Dong et al., 2006). In particular, *CLT4* and *TEP1* act against the malaria parasite as agonists and antagonists, respectively. *TEP1* together LRIM1 mediate the killing of ookinetes in the midgut epithelium; in contrast, *CTL4* and the C-type lectin CTLMA2, protect the parasite inhibiting its melanization (Osta et al., 2004).

The supplementation of different concentrations of *Asaia* cells on newly emerged females showed distinctive effects in *An. stephensi* and *An. gambiae*. In *An. stephensi*, where the bacterium is among the dominant symbionts, *Asaia* quickly reached a persistent and consistent homeostasis in every experimental group. In Asa4 and Asa8 groups, the fluctuations of the bacterial load at day 1, until reaching the homeostasis at day 3, could be correlated to the up-regulation of the *CTL4* gene, confirming its role in the regulation of the natural microbiota, in particular in relation to Gram-negative bacteria (Osta et al., 2004; Pang et al., 2016). *Asaia* density reached a constant plateau phase, remaining constant in both *Asaia*-challenged and control groups during later time points, possibly associated with the transcriptional induction of *CEC1* and *DEF1* genes.

In *An. gambiae, Asaia* showed to be differently regulated: likely its role as a secondary component of the natural microbiota of the African malaria vector could explain the difference in tolerance (Mancini et al., 2018). In fact, natural *Asaia* (sugar-fed control group) showed a gradual constant growth over time, while the infection with higher doses of cells underwent rapid fluctuations. This trend could be explained by the synergic action of the genes *CEC1*, *TEP1*, *DEF1* and *CTL4* in maintaining the microbiota balance. Concerning to the possible effect of bacterial challenges on *Plasmodium* infection, we have shown the ability of *Asaia* to activate the mosquito basal level immunity interfering with *Plasmodium* development *in vivo*. In fact, a significant reduction of malaria parasite load occurs five days after the infected blood meal, which coincides with stage when the parasite is present in midgut epithelium as premature oocysts (Wang and Jacobs-Lorena, 2013). The ability of *Asaia* to interfere with insect pathogens is corroborated by evidence in leafhopper as recently demonstrated by Gonella et al. (2019). The significant up-regulation of *TEP1* in *Asaia*-challenged *An. stephensi*, despite the bacterial concentrations, could be correlated to its decrease in vector competence and indicates an indirect and exploitable interplay between *Asaia* and the parasite. Indeed, the contribution of the microbiota of the different modulations of the mosquito immune response, seems to be correlated just to the strong presence of *Asaia* as demonstrated by the 16S Miseq analysis, showing it as the most abundant bacterium in mosquito administrated with *Asaia* enriched diets.

The reduction of the parasite load observed in *An. stephensi* was not conserved in *An. gambiae* where the lack of activation of *TEP1* in *Asaia*-challenged samples could explain the lack of *Plasmodium* inhibition in *An. gambiae*.

However, *An. gambiae* is not the natural vector of *P. berghei*, for which it is significantly more permissive than *P. falciparum* (Sinden et al., 2004). Moreover, *An. gambiae* mosquitoes showed a different transcriptional response to infection with *P. berghei* and *P. falciparum* (Dong et al., 2006). At light of consideration, further investigations on a possible *Asaia* role in immune stimulation in the system *An. gambiae*–*P. falciparum* are needed.

Nevertheless, these findings, while providing evidences on the ability of *Asaia* to stimulate the basal level of mosquito immunity in two main malaria vectors, and to naturally reduce the development of malaria parasite oocysts in *An. stephensi*. These findings confirm and expand its potential in SC approaches, not only through paratransgenesis, but also as a promising effector for mosquito immune priming.

## Data Availability

The raw data supporting the conclusions of this manuscript will be made available by the authors, without undue reservation, to any qualified researcher.

## Ethics Statement

All animal experiments were carried out according to the Italian Directive 116 of 10/27/92 on the “use and protection of laboratory animals” and in adherence with the European regulation (86/609) of 11/24/86, licence no. 125/94A, issued by the Italian Ministry of Health. The experiments were approved by the Ethic Committee of the University of Camerino (Protocol number 7/2014).

## Author Contributions

GF, MM, CD, and AC conceived and designed the experiments. GF supervised the project and gave conceptual advice. MM, CD, AC, MV, PR, AS, IR, and GF participated in the sample collection. MM, CD and AC performed the molecular analysis. GF and MM drafted the manuscript. GF, MM, CD, and AC edited the manuscript. All authors read and approved the final manuscript.

## Funding

This study was supported by Grant PRIN 2012 (2012T85B3R_001) and 2015 (2015JXC3JF_001) from Italian Ministry of Education, University and Research both to GF; Grant FAR, Fondo di Ateneo alla Ricerca 2014 from the University of Camerino to GF.

## Conflict of Interest Statement

The authors declare that the research was conducted in the absence of any commercial or financial relationships that could be construed as a potential conflict of interest.
