# Supplementary material for: Asaia Activates Immune Genes in Mosquito Eliciting an Anti-Plasmodium Response: Implications in Malaria Control
Source: Front Genet. 2019 Sep 25;10:836. doi: 10.3389/fgene.2019.00836 (PMC6774264; doi:10.3389/fgene.2019.00836)
Supplement: Supplementary file 1 [file Image_1.pdf]

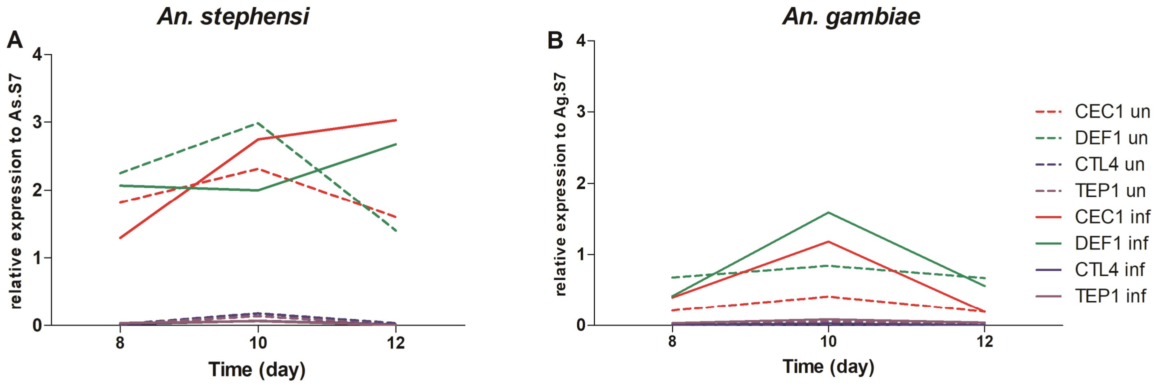

Fig. S1. Relative expression of immune genes in *An. stephensi* and *An. gambiae* administrated with sugar diet after uninfected and *P. berghei*-infected blood meal**.** Graphic representation of related modulation of different immune effectors after uninfected and infected blood meal (A and B) in mosquitoes fed on 5% sugar solution. Shown values represent the relative genes expression normalized on the reference gene Rps7. Scattered lines: uninfected blood meal, full lines: infected blood meal.
