# Supplementary material for: Asaia Activates Immune Genes in Mosquito Eliciting an Anti-Plasmodium Response: Implications in Malaria Control
Source: Front Genet. 2019 Sep 25;10:836. doi: 10.3389/fgene.2019.00836 (PMC6774264; doi:10.3389/fgene.2019.00836)
Supplement: Supplementary file 3 [file Image_3.pdf]

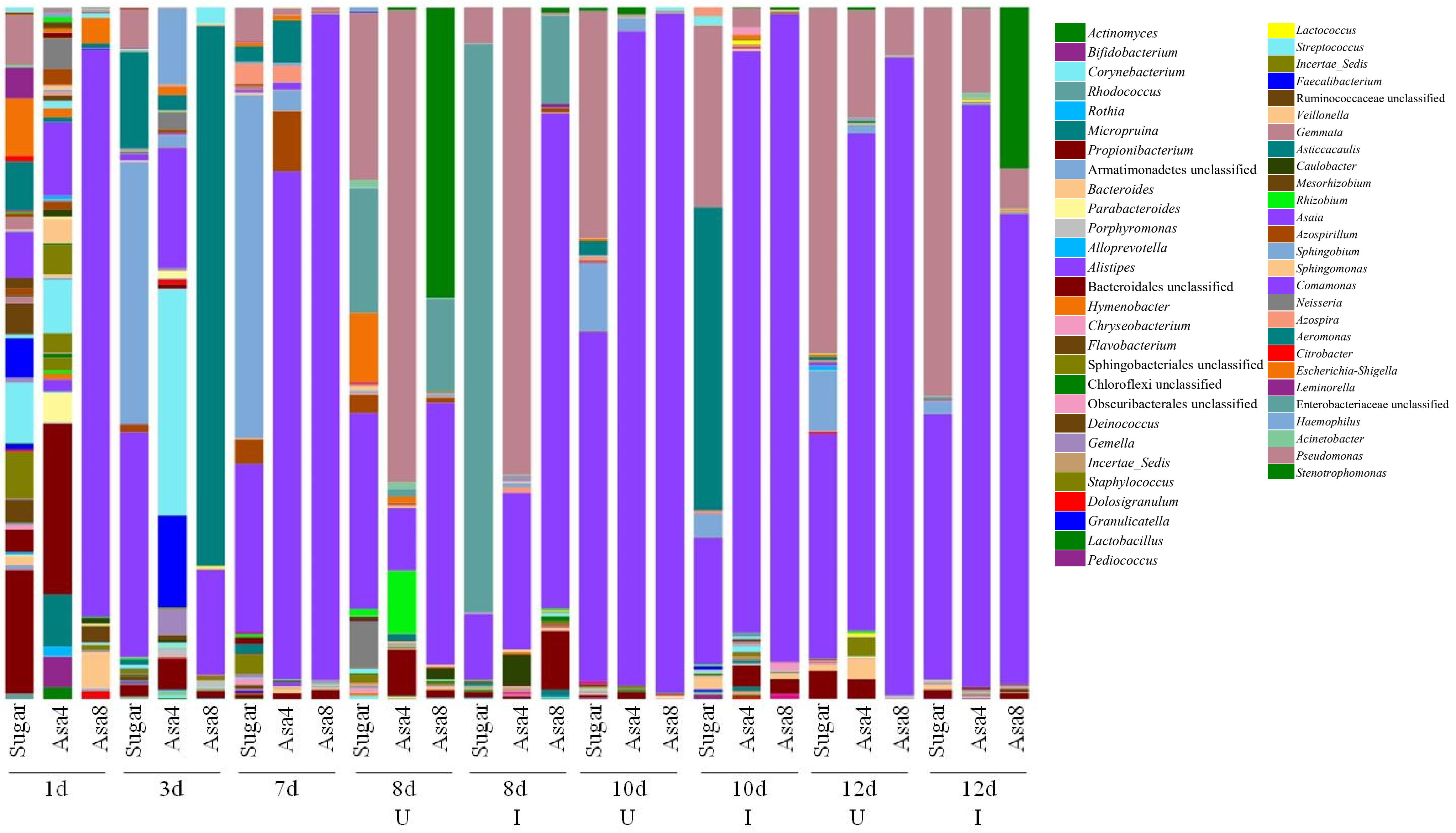

Fig. S3. Genus level composition (% of OTUs) in *An. stephensi* mosquitoes fed on different diet and different blood meal: uninfected (U) or infected (I).
