# Supplementary material for: Asaia Activates Immune Genes in Mosquito Eliciting an Anti-Plasmodium Response: Implications in Malaria Control
Source: Front Genet. 2019 Sep 25;10:836. doi: 10.3389/fgene.2019.00836 (PMC6774264; doi:10.3389/fgene.2019.00836)
Supplement: Supplementary file 4 [file Table_1.pdf]

Table S1: Sequences and efficiency of oligoncleotides used in quantitative RT-PCR to assess *Asaia* and *Plasmodium* density and the expression of immune genes *CEC1*, *DEF1*, *CTL4* and *TEP1*.

Gene	Forward (5'-3')	Reverse (5'-3')	Eff. %	Reference
Asaia	TAGCGTTGCTCGGAATGACTGG	CGTATCAAATGCAGCCCCAAGG	99.4	Capone et al. 2013
**Pl. 28S**	GTGGCCTATCGATCCTTTA	GCGTCCCAATGATAGGAAGA	97	Jaramillo-Gutierrez et al. 2009
An. stephensi				
**S7**	AGCAGCAGCAGCACTTGATTTG	TAAACGGCTTTCTGCGTCACCC	96.7	Capone et al. 2013
**DEF1**	GCGGTGGAGAACTATCGCGC	GCGATGCAATGCGCGGCAC	100.4	This work
**CECA**	GCCAGACGGAAGCGGGACG	CCACCACCGGGAGAGCTTTCTC	99.6	This work
**CTL4**	GATTGGAGCGAACACTCTTGCG	CGAAAGCTGATTACCGGTCGG	99.5	This work
**TEP1**	ACGGACAGCGAGGCTTTAGC	CTGAACCGTATCGCCCGG	99.6	This work
An. gambiae				
**S7**	AGAACCAGCAGACCACCATC	CTGCAAACTTCGGCTATTC	93.5	Kambris et al. 2009
**DEF1**	CATGCCGCGCTGGAGAACTA	GATAGCGGCGAGCGATACAGTGA	95	Kambris et al. 2009
**CEC1**	CCAGAGACCAACCAACCACCAA	GCACTGCCAGCACGACAAAGA	98.2	Kambris et al. 2009
**CTL4**	ATCGGAATGTCGATCGCTAC	GTGTCCGGCGATCAAACTAT	95.3	Kambris et al. 2009
**TEP1**	GTTCCAGGAGCGTACGTTGG	CCTGGCGAACAGACCCAAGCTG	99.9	Kambris et al. 2009

Capone, A., Ricci, I., Damiani, C., Mosca, M., Rossi, P., Scuppa, P. et al. (2013) Interactions between *Asaia*, *Plasmodium* and *Anopheles*: new insights into mosquito symbiosis and implications in malaria symbiotic control. *Parasit. Vectors* 6, 182. doi: 10.1186/1756-3305-6-182.

Jaramillo-Gutierrez, G., Rodrigues, J., Ndikuyeze, G., Povelones, M., Molina-Cruz, A., Barillas-Mury, C. (2009) Mosquito immune responses and compatibility between *Plasmodium* parasites and anopheline mosquitoes. *BMC Microbiol.* 9, 154. doi: 10.1186/1471-2180-9-154.

Kambris, Z., Cook, P.E., Phuc, H.K., Sinkins, S.P. (2009) Immune activation by life-shortening *Wolbachia* and reduced filarial competence in mosquitoes. *Science* 326, 134-136. doi: 10.1126/science.1177531
